# Copper‐Mediated Polymerization without External Deoxygenation or Oxygen Scavengers

**DOI:** 10.1002/anie.201804205

**Published:** 2018-06-19

**Authors:** Evelina Liarou, Richard Whitfield, Athina Anastasaki, Nikolaos G. Engelis, Glen R. Jones, Kelly Velonia, David M. Haddleton

**Affiliations:** ^1^ Department of Chemistry University of Warwick Library Road Coventry CV4 7AL UK; ^2^ Department of Materials Science and Technology University of Crete University Campus Voutes 71003 Heraklion Crete Greece

**Keywords:** ATRP, copper, monomers, oxygen, polymerization

## Abstract

As a method for overcoming the challenge of rigorous deoxygenation in copper‐mediated controlled radical polymerization processes [e.g., atom‐transfer radical polymerization (ATRP)], reported here is a simple Cu^0^‐RDRP (RDRP=reversible deactivation radical polymerization) system in the absence of external additives (e.g., reducing agents, enzymes etc.). By simply adjusting the headspace of the reaction vessel, a wide range of monomers, namely acrylates, methacrylates, acrylamides, and styrene, can be polymerized in a controlled manner to yield polymers with low dispersities, near‐quantitative conversions, and high end‐group fidelity. Significantly, this approach is scalable (ca. 125 g), tolerant to elevated temperatures, compatible with both organic and aqueous media, and does not rely on external stimuli which may limit the monomer pool. The robustness and versatility of this methodology is further demonstrated by the applicability to other copper‐mediated techniques, including conventional ATRP and light‐mediated approaches.

Among the various reversible deactivation radical polymerization (RDRP) methods, reversible addition‐fragmentation chain‐transfer polymerization (RAFT),^1^, ^2^, ^3^ atom‐transfer radical polymerization (ATRP),^4^, ^5^ and nitroxide‐mediated polymerization (NMP)^6^ are arguably the most popular, enabling the synthesis of polymeric materials with excellent control over molecular weight, functionality, dispersity, and architecture.^1^, ^7^, ^8^, ^9^ However, the integrity and precision of these materials can be compromised by potential oxygen contamination during the polymerization as it can irreversibly react with the reaction components (e.g. with initiator/macroinitiator, catalyst etc.), leading to terminated polymer chains and/or cessation of the polymerization.^10^ To avoid this contamination and eliminate oxygen from the polymerization mixture, costly and time‐consuming deoxygenation processes, such as freeze‐pump‐thaw and inert gas sparging, are typically employed. However, these methods can be incompatible with proteins/enzymes because of potential denaturation or loss of enzymatic activity, and require specialized equipment (e.g., Schlenk lines).^11^ In addition, the duration and rate of sparging may affect the concentration of volatile reagents, thereby leading to inconsistencies and inaccuracies. Importantly, the stringent anaerobic conditions required for most RDRP methods limit their potential applications.^12^

Recently, considerable interest has been directed towards oxygen‐tolerant polymerization methods aiming to simplify the polymerization protocol and eliminate the aforementioned deoxygenation techniques (Figure 1).^13^ For instance, Chapman et al. elegantly used enzymes, such as glucose oxidase (GOx), to effectively deoxygenate traditional RAFT polymerizations.^14^, ^15^ Boyer and co‐workers exploited photoinduced electron transfer (PET) RAFT to produce polymeric materials in open reaction vessels by either increasing the concentration of the photocatalyst or employing a reducing agent (e.g., ascorbic acid).^16^, ^17^, ^18^ Matyjaszewski and co‐workers employed initiators for continuous activator regeneration atom‐transfer radical polymerization (ICAR‐ATRP) and continuously converted oxygen into carbon dioxide by GOx catalysis, in the presence of sequential sacrificial substrates.^19^ Other groups also utilized ATRP, and variations thereof, to deoxygenate polymerization mixtures in the presence of external additives and reducing agents.^20^, ^21^, ^22^, ^23^, ^24^ Despite these great developments, the vast majority of the current approaches rely on either light activation or the use of additional reagents such as reducing agents and enzymes.^25^ However, photomediated methods can be incompatible with specific enzymes and proteins as their secondary structure can be disrupted through irradiation.^26^, ^27^, ^28^ In addition, utilizing light as an external stimuli may limit the monomer pool as strongly absorbing monomers, including chromophores, would be incompatible with these techniques.^29^, ^30^ Additionally, external reducing agents and enzymes can be costly, interfere with the monomer structure, be temperature dependent, or alter the pH of the polymerization mixture, thus significantly increasing the complexity of a given system.^31^, ^32^ Further limitations of the reported methods include the risk of generating additional chains through side products^19^ and the incompatibility with a wide range of monomers, temperatures, and solvents.^33^

**Figure 1:**
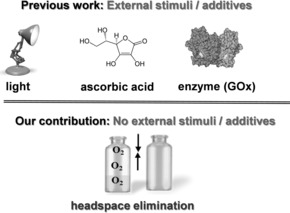
Oxygen‐tolerant approaches for RDRP.

To address these limitations we sought a simple system which would not rely on external stimuli or additional reagents to remove oxygen. We focused on Cu^0^‐wire‐mediated RDRP, a system consisting of a number of components which could play the role of the reducing agent/oxygen scavenger such as the initiator, the N‐containing ligand, and the Cu^0^ wire which can consume oxygen by oxidation into Cu^I^ or Cu^II^.^7^ Initial experiments involved preparing a Cu^0^‐wire‐catalyzed polymerization in a 28 mL unsealed vial, wherein the total reaction volume was 8 mL, with methyl acrylate (targeting DP_*n*_=50) as the monomer, ethyl α‐bromoisobutyrate (EBiB) as the initiator, tris(2‐(dimethylamino)ethyl)amine (Me_6_Tren) as the ligand, and dimethyl sulfoxide (DMSO) as the solvent, in the absence of any commonly employed deoxygenation procedures (i.e., nitrogen sparging or freeze‐pump‐thaw). No polymerization took place, even when the reaction was left to proceed for more than 48 hours. However, upon sealing the vial with a septum (or a screw cap; see Figure S1a in the Supporting Information) polymerization reached near‐quantitative conversion within 11 hours, thereby achieving dispersities as low as 1.10. This data supports our hypothesis that we have a “self‐degassing” system. Importantly, and despite the narrow molecular‐weight distributions observed, the experimental molecular weight (*M*_n_=6600) deviated significantly from the theoretical value (*M*_n_=4500), and this was attributed to low initiator efficiency (see Figure S2a–c). To clarify this, we also performed an identical experiment where freeze‐pump‐thaw cycles were used to thoroughly deoxygenate the reaction mixture prior to polymerization (see Figure S3a, Table 1, and Table S1a). In agreement with the literature,^34^ much lower molecular weights were achieved (*M*_n_=5300), suggesting that part of the initiator is consumed during the early stages of the polymerization, leading to higher than expected molecular weights. This data implied that under the reaction conditions studied, the initiator is somehow acting as an oxygen scavenger prior to the polymerization.

**Table 1:** ^1^H NMR and SEC analyses for PMA (targeted DP_*n*_=50) with different headspace volumes.^[a]^

Headspace [mL]	*t* ^[b]^ [h]	Deoxygenation Process	*M*_n, th._ [g mol^−1^]	*M*_n, SEC_^[c]^	*Đ*
–	4	FPT	4400	5300	1.08
0	4	none	4300	5200	1.07
12	6	none	4300	6200	1.07
20	11	none	4300	6600	1.10

[a] [MA]/[EBiB]/[CuBr_2_]/[Me_6_Tren]=50:1:0.05:0.18 in DMSO (50 %, v/v) solvent. [b] Based on kinetics (see Figure S4 and Table S1b the Supporting Information). [c] Determined by THF SEC analysis and expressed as molecular‐weight equivalents to PMMA standards (see Figure S1b and Table S1a). FPT=Freeze‐pump‐thaw.

We envisaged that by reducing the headspace within the vial, the amount of oxygen would also be reduced, leading to improved initiator efficiencies. Indeed, by maintaining the reaction volume constant at 8 mL and altering the size of the vial from 28 mL (20 mL of headspace) to 20 mL (12 mL of headspace) and 8 mL (no headspace), the initiator efficiency was significantly improved, yielding polymers with *M*_n_=6200 and 5200, respectively (see Figures S1b and S4, Table 1, and Table S1a–S1c). Thus, in the absence of any deoxygenation procedures and by simply eliminating the headspace within the vessel, similar initiator efficiencies, rates of reaction, and control over the polymerization, in comparison to the externally degassed system, were achieved. The synthetic ease of this approach was further demonstrated by performing the polymerization on a multigram scale (ca. 125 g) with well‐defined poly(MA) obtained (*Đ*≈1.10) in high yields (>90 % conversion; see Figure S5).

To explore the utility of this system across a wide range of molar masses, we investigated the ability to target higher degrees of polymerization. Under otherwise identical reaction conditions, targeting DP_*n*_=100–1000 resulted in high conversions (89–97 %), low *Đ* values (1.06–1.13), and good agreement between theoretical and experimental molecular weights (see Figure S6 and Table S2). It should be noted that for higher targeted molecular weights, longer reaction times were required, as expected. With these reaction conditions, the polymerization was screened in a selection of organic solvents including acetonitrile, toluene, methanol, isopropanol, and trifluoroethanol. In all cases, well‐defined polymers with low dispersities and high yields were obtained (see Figure S7 and Table S3). Importantly, this approach was effective in both homogeneous (e.g., hexyl acrylate in TFE; see Figure S10) and heterogeneous/biphasic systems (e.g., butyl acrylate in DMSO; see Figure S8)^35^ with the same level of control, highlighting the robustness of this system. Finally, when water was employed as the solvent (utilizing PEGA instead of MA) and upon slightly optimizing the reaction conditions (see the Supporting Information and Figure S9), well‐defined poly(PEGA) was obtained with low final dispersities (*Đ*≈1.2), thus expanding the scope to include both organic and aqueous media.

Additional monomer families were also investigated. Using previously established polymerization protocols,^34^, ^36^, ^37^ acrylates (Figure 2; see Figures S8–S10), methacrylates (see Figure S11), acrylamides (see Figure S12), and styrene (see Figure S13) were successfully polymerized and yielded well‐controlled polymers with narrow molecular‐weight distributions in the absence of any standard deoxygenation protocols. To the best of our knowledge, this is the first time that four different monomer families can be polymerized through an oxygen‐tolerant copper‐mediated methodology. A fundamental requirement of a controlled polymerization is the retention of active chain‐ends. The chain‐end fidelity for the PMA was determined by analysis of a low‐molecular‐weight sample (DP_*n*_=25). Matrix assisted laser desorption‐ionization time‐of‐flight mass spectrometry (MALDI‐ToF‐MS) revealed a single peak distribution corresponding to *m*/*z* values for polymer chains comprising of the expected chain‐ends, initiated with EBiB and capped by bromine (Figure 2 a).

**Figure 2:**
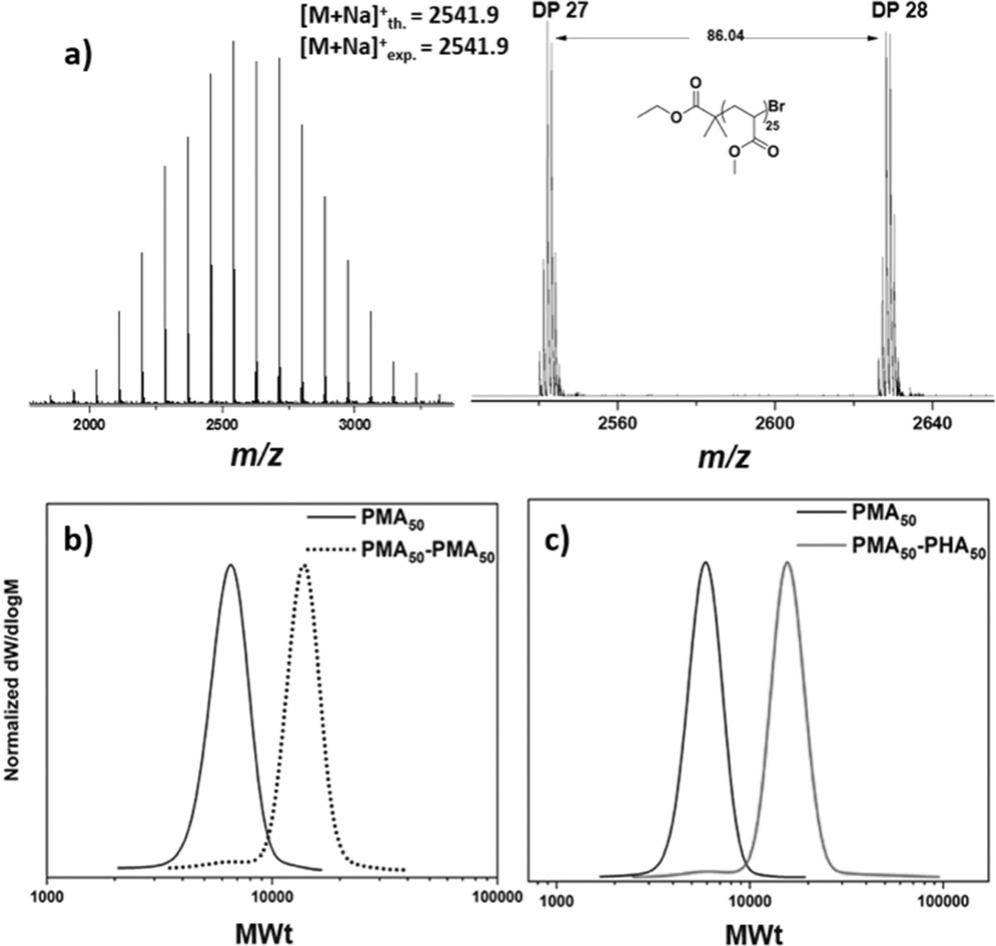
a) MALDI‐ToF‐MS spectra of PMA (DP_*n*_=25) synthesized in the absence of external deoxygenation procedures. SEC traces for PMA_50_‐PMA_50_ (b) and PMA_50_‐*b*‐PHA_50_ (c)

Characterization by ^1^H NMR spectroscopy also confirmed the bromine ω‐functionality to be close to 100 % when comparing signals corresponding to the ω‐terminal methine signal with the CH_3_ groups of the isobutyrate group of EBiB. The synthetic utility of these chain ends was then explored by in situ re‐initiation of the macroinitiator with a second aliquot of MA (Figure 2 b; see Table S4).

Although a clear shift to higher molecular weights was observed, a small low‐molecular‐weight shoulder was evident by SEC, indicating some termination events. This observation was attributed to the introduction of additional dissolved oxygen with the second monomer aliquot which was then responsible for the termination of propagating radicals. To verify this, the synthesis of the first poly(MA) block was repeated as previously, in the absence of any freeze‐pump‐thaw or nitrogen sparging. Upon reaching near‐quantitative conversion (>97 %), a second aliquot of deoxygenated MA was then added (see Figure S14). In this case, very good control was observed with the molecular weight distribution completely shifting to higher molecular weights and a final dispersity as low as 1.06. This data suggests that the end‐group fidelity of the initial block was indeed close to 100 % prior to the addition of the second monomer and that it is the dissolved oxygen that is responsible for the observed small amount of termination.

To further investigate the consumption of oxygen, we conducted experiments with an optical oxygen sensor, thus enabling online monitoring of the dissolved oxygen concentration in the polymerization mixtures (see Figure S15). In the presence of bigger headspaces (i.e., 20 and 12 mL), the oxygen consumption was slow, requiring one hour to reach about 2 mg L^−1^ and 0.8 mg L^−1^, respectively (typical initial dissolved oxygen concentration is ca. 7.5 mg L^−1^). On the contrary, upon eliminating the headspace, the oxygen was rapidly consumed within 5 minutes (ca. 0 mg L^−1^), which explains the shorter reaction times observed for this system (ca. 2 h for the polymerization to reach completion) in comparison to those with the increased headspace (6–11 h to reach completion; Figure 3 a). To better understand which component is responsible for the rapid oxygen consumption we first prepared a polymerization mixture with MA, DMSO, CuBr_2_, and Me_6_Tren. In the absence of Cu^0^ wire and initiator very little, if any, oxygen consumption was observed within one 1 hour, thus suggesting that the ligand had very limited reactivity with oxygen. However, in the absence of initiator (only Cu^0^ wire present) a complete oxygen consumption took place in 42 minutes, thus highlighting the capability of Cu^0^ wire to act as a reducing agent. As such, these experiments suggest that the initiator is prominently participating in the oxygen consumption (Figure 3 b). This observation is further supported by the lower initiator efficiency observed in the presence of bigger headspaces (see Figures S2a–c and Table S4), the longer reaction times when targeting polymers of higher molecular weights (lower concentration of initiator would lead to slower oxygen consumption), and by the incapability of our system to afford “perfect” in situ block copolymers. Nevertheless, when both Cu^0^ wire and initiator were present, the oxygen was consumed within 5 minutes (twice as fast as when only initiator was present), which indicates that it is the combined presence of initiator and wire that leads to complete oxygen consumption. The detailed mechanism of this reaction is currently under investigation and will be the subject of a forthcoming publication.

**Figure 3:**
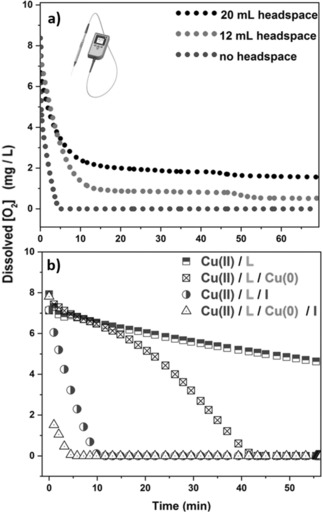
Line graphs illustrating a) the effect of the headspace and b) the effects of Cu^0^ wire, EBiB (I), and Me_6_Tren (L) on the evolution of the dissolved oxygen concentration during polymerization.

Based on our findings we envisaged that our approach may be compatible with a number of additional copper mediated protocols. Indeed, conventional (or normal) ATRP^38^ (when only CuBr is employed) of MMA was subsequently conducted at 90 °C. By eliminating the headspace and in the absence of any external deoxygenation methods, PMMA with low dispersity and high yields was obtained (see Figure S16). For applications where spatiotemporal control is required, photomediated methodologies are typically utilized.^39^, ^40^ Pleasingly, the employment of photomediated polymerization presented well‐defined polymers, thus further highlighting the versatility and robustness of this methodology (see Figure S17).

In summary, we report a facile, efficacious, robust, and versatile method, which avoids rigorous deoxygenation in controlled radical polymerization by simply eliminating the reaction vessel's headspace. Well‐defined polymers consisting of different monomer families were obtained in a controlled manner with high end‐group fidelity. The user‐friendly nature of our approach expands the current scope of oxygen‐tolerant polymerization strategies and offers a unique synthetic platform for the preparation of well‐defined materials.

## Conflict of interest

The authors declare no conflict of interest.

## Supporting information

As a service to our authors and readers, this journal provides supporting information supplied by the authors. Such materials are peer reviewed and may be re‐organized for online delivery, but are not copy‐edited or typeset. Technical support issues arising from supporting information (other than missing files) should be addressed to the authors.

Supplementary
