# Supplementary material for: Copper‐Mediated Polymerization without External Deoxygenation or Oxygen Scavengers
Source: Angew Chem Int Ed Engl. 2018 Jun 19;57(29):8998–9002. doi: 10.1002/anie.201804205 (PMC6055709; doi:10.1002/anie.201804205)
Supplement: Supplementary file 1 — Supplementary [file ANIE-57-8998-s001.pdf]

Angewandte Chemie
Eine Zeitschrift der Gesellschaft Deutscher Chemiker

Supporting Information

Copper-Mediated Polymerization without External Deoxygenation or Oxygen Scavengers

Evelina Liarou+, Richard Whitfield+, Athina Anastasaki, Nikolaos G. Engelis, Glen R. Jones, Kelly Velonia, and David M. Haddleton**

anie_201804205_sm_miscellaneous_information.pdf

Author Contributions

R.W. Investigation: Equal
N.E. Investigation: Supporting
G.J. Investigation: Supporting
K.V. Investigation: Supporting.

Table of Contents

Experimental 2
Instrumentation........ 2
General Procedures 3
Results and Discussion........ 5
References 16
Author Contributions........ 16

Experimental

All materials were purchased from Sigma-Aldrich or Fischer Scientific and used as received unless otherwise stated. Copper(I) bromide (CuBr, 98%, Sigma-Aldrich) was washed with acetic acid and ethanol and dried under vacuum prior to use. Copper(0) in the form of wire (gauge 0.25 mm) was purchased from Comax Engineered wires and purified by immersion in concentrated 37 % HCl for 15 minutes, subsequently rinsed with distilled water and acetone, and dried with compressed air prior to use. Tris-(2-(dimethylamino)ethyl)amine (Me_6Tren) was synthesized according to the literature and stored in the fridge.[1] *N,N,N',N'',N''*-pentamethyldiethylenetriamine (PMDETA) was distilled prior to use. *N*-(*n*-Propyl)-2-pyridylmethanimine was synthesized according to literature procedure and stored under a nitrogen atmosphere prior to use.[2] Water soluble initiator 2,3-dihydroxypropyl 2-bromo-2-methylpropanoate was synthesized according to literature procedure.[3]

Instrumentation

^{1}H NMR.
^{1}H NMR spectra were recorded on Bruker DPX-300 or DPX-400 spectrometers in deuterated chloroform ($CDCl_3$) or deuterium oxide (D_2O) obtained from Sigma-Aldrich. Chemical shifts are given in ppm downfield from the internal standard tetramethylsilane. Monomer conversions were determined via ^{1}H NMR spectroscopy by comparing the integrals of monomeric vinyl protons to polymer signals.

Size Exclusion Chromatography.
THF. SEC measurements were carried out with an Agilent 390-LC MDS instrument equipped with differential refractive index (DRI), viscometry (VS), dual angle light scatter (LS) and dual wavelength UV detectors. The system was equipped with 2 x PLgel Mixed C columns (300 x 7.5 mm) and a PLgel 5 µm guard column. The eluent was THF with 2 % TEA (triethylamine) and 0.01 % BHT (butylated hydroxytoluene) additives. Samples were run at 1 mL / min at 30ºC. Poly(methyl methacrylate) was used as a standard in all cases (Agilent EasyVials) to create a third order calibration between 550 gmol^{-1} and 1,568,000 gmol^{-1}, except in the analysis of PS where polystyrene calibrants were utilized. Analyte samples were filtered through a GVHP membrane with 0.22 *µ*m pore size before injection. Respectively, experimental molar mass ($M_{n,SEC}$) and dispersity (*Đ*) values of synthesized polymers were determined by conventional calibration using Agilent GPC/SEC software (version A.02.01).
DMF. An Agilent 390-LC MDS instrument equipped with differential refractive index (DRI), viscometry (VS), dual angle light scatter (LS) and UV detectors was equipped with 2 x PLgel Mixed D columns (300 x 7.5 mm) and a PLgel 5 µm guard column. The eluent was DMF with 5 mmol NH_4BF_4 additive. Samples were run at 1mL/min at 50ºC. Poly(methyl methacrylate) standards (Agilent EasyVials) were used to create a third order calibration between 550 gmol^{-1} and 955,000 gmol^{-1}. Analyte samples were filtered through a nylon membrane with 0.22 µm pore size before injection. Respectively, experimental molar mass (M_n,SEC) and dispersity (*Đ*) values of synthesized polymers were determined by conventional calibration and universal calibration using Agilent GPC/SEC software (version A.02.01).

MALDI-ToF-MS.
MALDI-ToF-MS measurements were conducted using a Bruker Daltonics Ultraflex II MALDI-ToF mass spectrometer, equipped with a nitrogen laser delivering 2 ns laser pulses at 337 nm with positive ion ToF detection performed using an accelerating voltage of 25 kV. Solutions in tetrahydrofuran (THF) (50 µL) of *trans*-2-[3-(4-*tert*-butylphenyl)-2-methyl-2-propyldene] malononitrile (DCTB) as a matrix (saturated solution), sodium iodide as the cationization agent (1.0 mg mL^{-1}) and sample (1.0 mg mL^{-1}) were mixed, and 0.7 µL of the mixture was applied to the target plate. Spectra were recorded in reflectron mode calibrated with Poly(ethylene glycol) monomethyl ether (PEG-Me) 1900 kDa.

Oxygen Probe.
Pocket Oxygen Meter - FireStingGO2 (from Pyro Science)**:** The solvent-resistant oxygen probe OXSOLV measures oxygen partial pressure in most polar and nonpolar solvents. It is based on optical detection principles (REDFLASH technology) and can be used both in pure and complex organic solvents. The fiber-optic oxygen sensor tip is covered with a stainless steel tube 1.5 mm in diameter and 150 (or 40) mm in length. The analysis of the data was conducted with the FireStingGO2 Manager software.

UV Source.
A UV nail gel curing lamp (λ_{max} ~ 365 nm) with four 9 Watt bulbs was used.

General Procedures

Cu(0)-wire mediated RDRP of acrylates

General procedure for the Cu(0)-mediated RDRP of methyl acrylate (MA) with targeted DP_n=50 (PMA_{50}) in the absence of any external deoxygenation.

MA (4 mL, 50 eq.), Me_6Tren (42.5 μL, 0.18 eq.), EBiB (129 μL, 1 eq.), Cu(II)Br_2 (9.86 mg, 0.05 eq.), DMSO (4 mL) and pre-activated copper wire (5 cm) wrapped around a stirring bar were added to septum sealed vials of 8 mL (no headspace left), 20 mL (12 mL headspace) and 28 mL (20 mL headspace). The polymerization was allowed to commence at ambient temperature. Kinetic studies were conducted to determine the time needed for near quantitative conversion to be reached. Once this conversion had been achieved, a sample was taken and passed through a short column of neutral alumina to remove dissolved copper salts prior to analysis by ^1H NMR in $CDCl_3$ and SEC in THF.

General procedure for the Cu(0)-mediated RDRP of *n*-butyl acrylate (BA) with targeted DP_n=50 (PBA_{50}) in the absence of any external deoxygenation.

BA (4 mL, 50 eq.), Me_6Tren (26.8 μL, 0.18 eq.), EBiB (81.9 μL, 1 eq.), Cu(II)Br_2 (6.25 mg, 0.05 eq.), DMSO (4 mL) and pre-activated copper wire (5 cm) wrapped around a stirring bar were added to a septum sealed vial. The polymerization was allowed to commence at RT. Kinetic studies were conducted to determine the time needed for quantitative conversion (typically >96%) and after the completion of the polymerization, a sample was taken and passed through a short column of neutral alumina to remove dissolved copper salts prior to analysis by ^1H NMR in $CDCl_3$ and SEC in THF.

General procedure for the Cu(0)-mediated RDRP of $PEGA_{480}$ with targeted DP_n=10 in the absence of any external deoxygenation.

HPLC grade H_2O (4 mL), Me_6Tren (212 μL, 0.792 mmol, 0.18 eq.) and Cu(II)Br_2 (49 mg, 0.22 mmol, 0.05 eq.) were charged to a 8 mL vial and vortexed until complete dissolution of Cu(II)Br_2. EBiB (645 μL, 4.4 mmol, 1 eq.), poly(ethylene glycol) methyl ether acrylate (average M_n 480) ($PEGA_{480}$) (4 mL, 44 mmol, 10 eq.) and pre-activated copper wire (5 cm) wrapped around a stirring bar were added to the vial. The vial sealed with a septum and the polymerization left to commence. Samples were taken periodically and conversions were measured using ^1H NMR in D_2O and SEC analysis in THF, after having been passed through neutral alumina.

General procedure for the Cu(0)-mediated RDRP of hexyl acrylate (HA) with DP_n=50 (PHA_{50}) in the absence of any external deoxygenation.

HA (4 mL, 50 eq.), Me_6Tren (22 μL, 0.18 eq.), EBiB (67 μL, 1 eq.), Cu(II)Br_2 (5.10 mg, 0.05 eq.), TFE (4 mL) and pre-activated copper wire (5 cm) wrapped around a stirring bar were added to an 8 mL septum sealed vial. The polymerization was allowed to commence at RT. Kinetic studies were conducted to determine the time needed for quantitative conversion (tyrically >96%) and after the completion of the polymerization, a sample was taken and passed through a short column of neutral alumina to remove dissolved copper salts prior to analysis by ^1H NMR in $CDCl_3$ and SEC in THF.

Cu(0)-wire mediated RDRP of methacrylates, acrylamides and styrene

General procedure for the Cu(0)-mediated RDRP of methyl methacrylate (MMA) with DP_n= 50 ($PMMA_{50}$) in the absence of external deoxygenation.

MMA (4 mL, 50 eq.), methyl-*α*-bromophenylacetate (MBPA) (119 μL, 1 eq.), Cu(II)Br_2 (8.35 mg, 0.05 eq.), DMSO (4 mL), Me_6Tren (36 μL, 0.18 eq.) and pre-activated copper wire (5 cm) wrapped around a stirring bar were added to an 8 mL septum sealed vial. The polymerization was allowed to commence at ambient temperature for 18 h without employing any deoxygenation procedure. After 18h, a sample dissolved in THF, passed through a short column of neutral alumina to remove dissolved copper salts prior to analysis by ^1H NMR in $CDCl_3$ and SEC in THF.

General procedure for the Cu(0)-mediated RDRP of *N*-isopropylacrylamide (NiPAm) (targeted DP_n=50) in the absence of any external deoxygenation procedure.

In a 8 mL vial, Cu(II)Br_2 (7.9 mg, 0.0353 mmol, 0.2 eq.), 1 mL HPLC grade H_2O and Me_6Tren (18.9 μL, 0.071 mmol, 0.4 eq.) were added and vortexed until total dissolution of Cu(II)Br_2. Simultaneoulsy, in another vial, NiPAm (1 g, 8.837 mmol, 50 eq.) was dissolved in HPLC grade H_2O (6 mL) after sonication (~10 min) and transferred in the 8 mL vial. The water soluble initiator 3-dihydroxypropyl 2-bromo-2-methylpropanoate (42.6 mg, 0.177 mmol, 1 eq.) after dissolution in 1 mL HPLC grade H_2O, was added in the 8 mL vial along with the pre-activated copper wire (140 cm) wrapped around a stirring bar . The vial was sealed with septum and the polymerization left to commence (in ice bath for the first hour). After 3 h the reaction was sampled, conversion was measured using ^1H NMR in D_2O and SEC analysis in DMF, after having been passed through a short neutral alumina column.

General procedure for the Cu(0)-mediated RDRP of styrene (St) with targeted DP_n=50 in the absence of any external deoxygenation.

Styrene (4 mL, 50 eq.), MBPA (0.111 mL, 1 eq.), $CuBr_2$ (7.80 mg, 0.05 eq.), IPA (4 mL), PMDETA (0.052 mL, 0.36 eq.) and pre-activated copper wire (5 cm) wrapped around a stirring bar were added to a septum sealed vial. The polymerization was allowed to commence at 60 ˚C for 36 h. After 36 h, a sample was taken and passed through a short column of neutral alumina to remove dissolved copper salts prior to analysis by ^{1}H NMR in $CDCl_3$ and SEC in THF.

Chain Extensions and block copolymers *via* Cu(0)-mediated RDRP

General procedure for the *in-situ* chain extension reaction for the synthesis of PMA_{50}-PMA_{50}.

MA (4 mL, 50 eq), DMSO (4 mL), EBiB (129 µL, 1 eq), Cu(II)Br_2 (9.86 mg, 0.05 eq), Me_6Tren (42.5 µL, 0.18 eq.) and pre-activated copper wire (5 cm) were added to a 20 mL septum sealed vial and the polymerization was allowed to commence. Upon reaching high conversion (>96%) for the first block of the homopolymer, a 1 : 1 (v/v) mixture of MA (4 mL) and DMSO (4 mL) was added to the reaction mixture and the polymerization was left to commence. Samples were taken periodically and conversions were measured using ^{1}H NMR in $CDCl_3$ and SEC analysis in THF, after having been being passed through neutral alumina.

General procedure for the synthesis of the block copolymer PMA_{50}-*b*-PHA_{50}.

The general procedure for Cu(0)-RDRP of MA was followed. Upon detection of >96% conversion a 1 : 1 (v/v) mixture of HA (4 mL) and DMSO (4 mL) was added to the reaction mixture without employing any deoxygenation process or nitrogen blanket. Utilizing a degassed syringe, samples were taken periodically for the measurement of conversion through ^{1}H NMR in $CDCl_3$ and SEC analysis in THF.

ATRP and photo-induced RDRP

General procedure for the ATRP of poly(methyl methacrylate) with targeted DP_n= 100 in the absence of external deoxygenation.[2]

For PMMA with a targeted DP_n=100, CuBr (0.134 g, 0.935 mmol), 9 mL toluene, *N*-(*n*-Propyl)-2-pyridylmethanimine (2 mol eq. to CuBr) and MMA (10 mL, 93.5 mmol) were added in a 20 mL vial immersed in a thermostated oil bath at 90 °C. When the contents reached reaction temperature, EBiB (0.137 mL, 0.935 mmol) was added. The polymerization was left to commence and samples were taken periodically for the measurement of conversion through ^{1}H NMR in $CDCl_3$ and SEC analysis in THF, after having been passed through neutral alumina for the removal of dissolved copper salts.

General procedure for the photo-induced copper-mediated RDRP of MA (targeted DP_n=50) in the absence of external deoxygenation.

MA (4 mL, 50 eq.), EBiB (129 µL, 1 eq.), Cu(II)Br_2 (4.0 mg, 0.02 eq.), Me_6Tren (28.3 µL, 0.12 eq.) and DMSO (4 mL) were added to a septum sealed vial and the polymerization was allowed to commence under a UV lamp (λ_{max} ~ 360 nm). Samples were taken periodically and conversions were measured using ^{1}H NMR in $CDCl_3$ and SEC analysis in THF, after having been passed through neutral alumina.

Results and Discussion

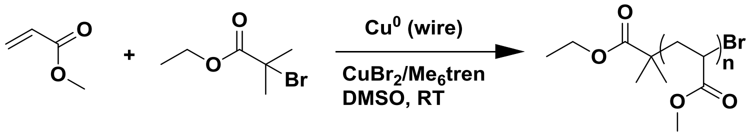

Scheme S1. Reaction scheme for the Cu(0)-mediated RDRP of PMA_{50}.

Table S1a. Detailed ^{1}H NMR and SEC analyses for PMA (targeted DP_n= 50) after freeze-pump-thaw deoxygenation, N_2 blanket in the headspace and without external deoxygenation with different headspace volumes.

Vessel	Headspace (mL)	Time (h)	Deoxygenation Process	Conversion (%)	$M_{n,\ th.}$ (g mol^{-1})	$M_{n,\ SEC}$[a]	*Đ*
Schlenk tube	-	4	3 x Freeze Pump Thaw cycles	98	4400	5300	1.08
20 mL vial	Filled with N_2	4	N_2 blanket	98	4400	5400	1.08
8 mL vial	0	4	None	96	4300	5200	1.07
20mL vial	12	6	None	96	4300	6200	1.07
28 mL vial	20	11	None	96	4300	6600	1.10

[a] Molecular Weight equivalents to PMMA.

Figure S1a. SEC trace for PMA_{50} with the vial sealed utilizing a lid yielding M_n= 5200[a], $Đ$ =1.10, conversion 93%, [MA]:[EBiB]:[$CuBr_2$]:[Me_6Tren] = [50]:[1]:[0.05]:[0.18]. DMSO as solvent 50% *v/v.* ([a] Molecular Weight equivalents to PMMA).

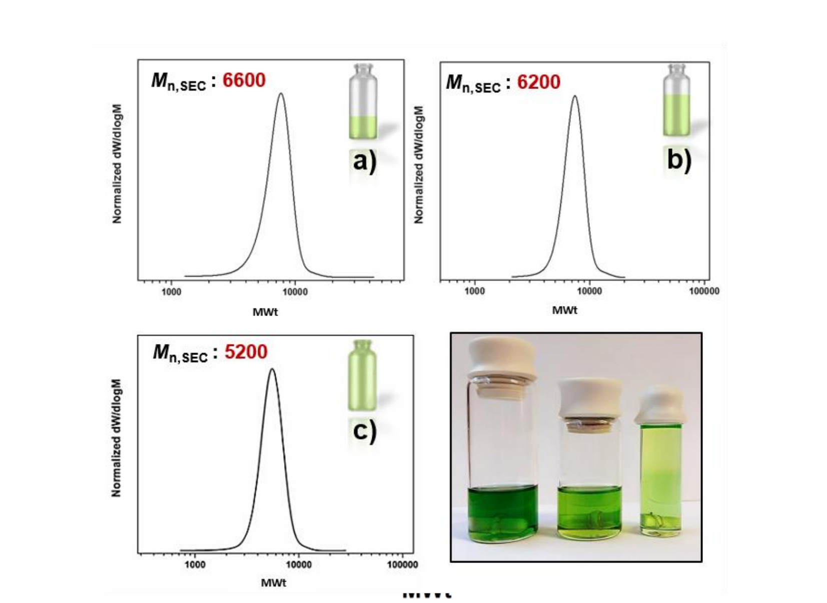

Figure S1b. **a), b) and c)** SEC traces for PMA_{50} in different vial sizes with [MA]:[EBiB]:[$CuBr_2$]:[Me_6Tren] = [50]:[1]:[0.05]:[0.18]. DMSO as solvent 50% v/v and **d)** their colour differences attributed to the generation of higher amounts of Cu(II) by either the oxidation of copper wire, or the consumption of the initiator radicals at the initial stage of the polymerization.

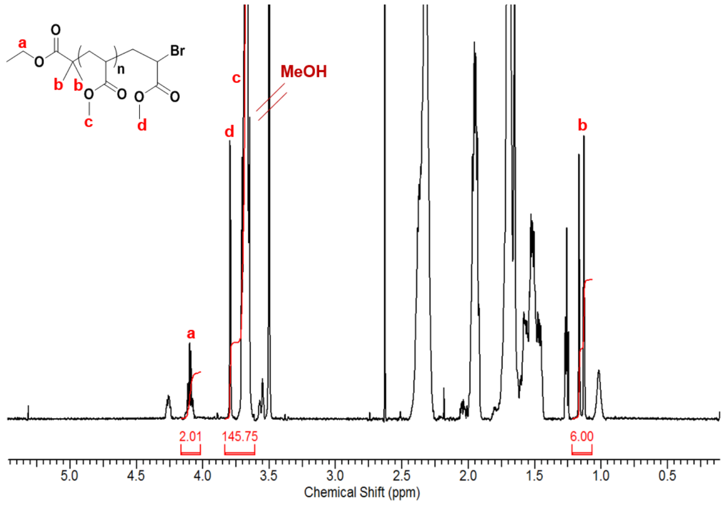

Figure S2a: ^{1}H NMR spectrum of the purified PMA_{50} synthesized via Cu(0)-RDRP in the absence of external deoxygenation, without headspace.

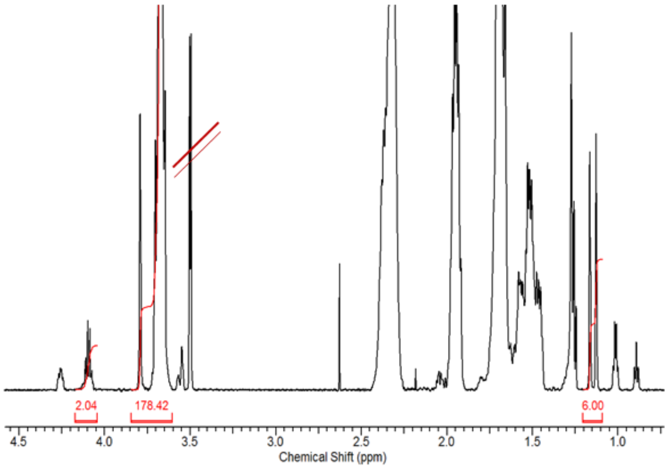

Figure S2b: ^{1}H NMR spectrum of the purified PMA_{50} synthesized via Cu(0)-RDRP in the absence of external deoxygenation, with 12 mL of headspace.

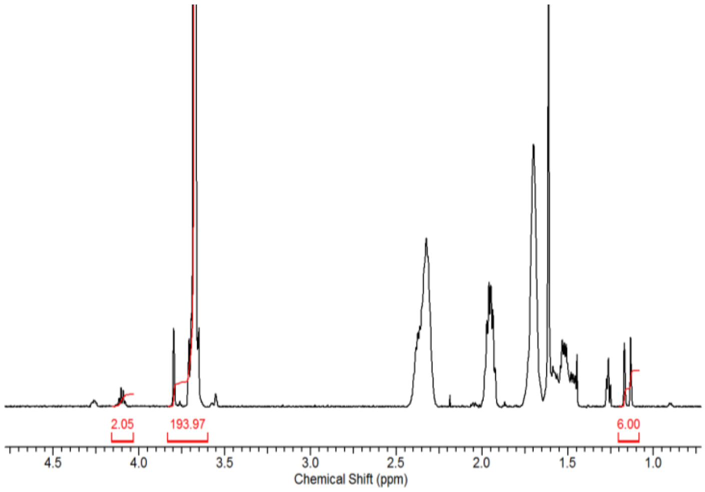

Figure S2c: ^{1}H NMR spectrum of the purified PMA_{50} synthesized via Cu(0)-RDRP in the absence of external deoxygenation, with 20 mL of headspace.

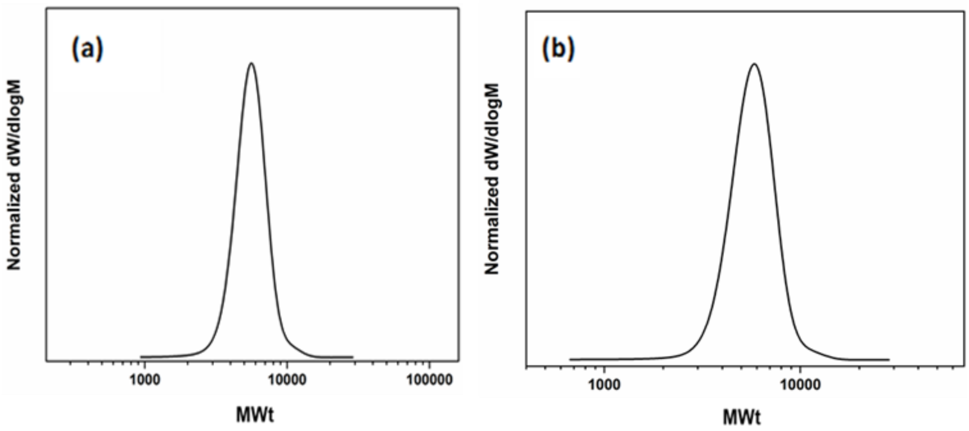

Figure S3. SEC traces for PMA_{50} (**a**) deoxygenated with 3 freeze-pump-thaw cycles and (b) the headspace of the vial filled with N_2.

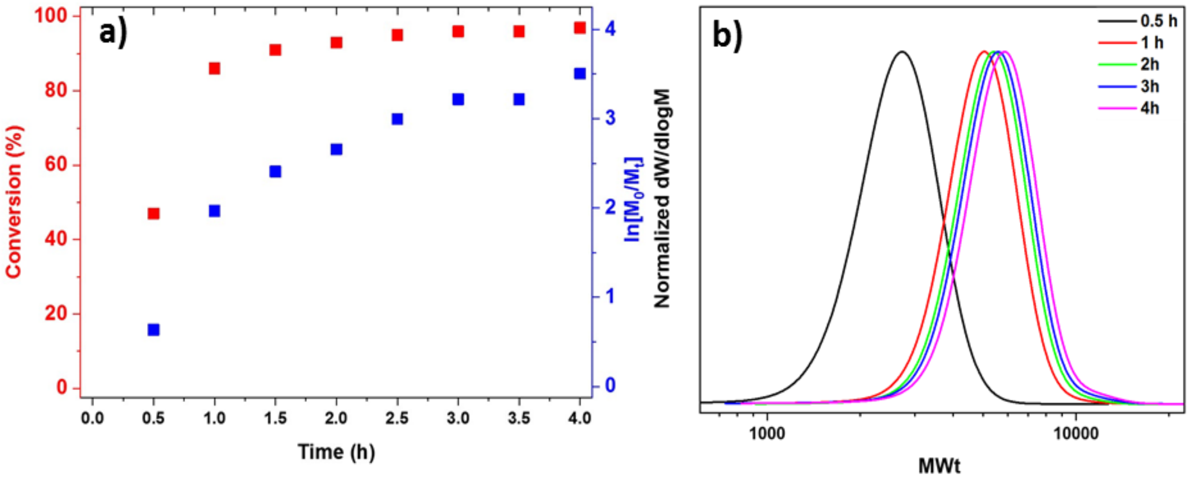

Figure S4. Deoxygenation-free polymerization kinetics for PMA_{50} in the absence of headspace with a) conversion and ln[M_0/M_t] versus time and b) SEC traces illustrated.

Table S1b. ^{1}H NMR and SEC analysis for the Cu(0)-mediated RDRP kinetics of MA with DP_n=50 (in the absence of external deoxygenation, without headspace).

Time (hrs)	Conversion (%)	$M_{n,\ th.}$ (g/mol)	$M_{n,\ SEC}$ [a]	$Đ$
0.5	47	2100	2500	1.09
1	86	3900	4700	1.07
1.5	91	4100	5100	1.07
2	93	4200	5100	1.08
2.5	95	4300	5300	1.08
3	96	4300	5300	1.08
3.5	96	4300	5300	1.08
4	97	4400	5500	1.08

[a] Molecular Weight equivalents to PMMA.

Table S1c: Relationship between moles of oxygen, M_n values and initiator efficiencies for PMA_{50} with different headspace volumes in the vials.

Headspace (mL)	Moles of Oxygen in headspace	$M_{n,\ theoretical}$ (g mol^{-1})	$M_{n,\ NMR}$ (g mol^{-1})	Initiator Efficiency (%)
0	~ 0	4300	4100	100
12	5.36×10^{-4}	4300	5100	84
20	8.93×10^{-4}	4200	5400	78

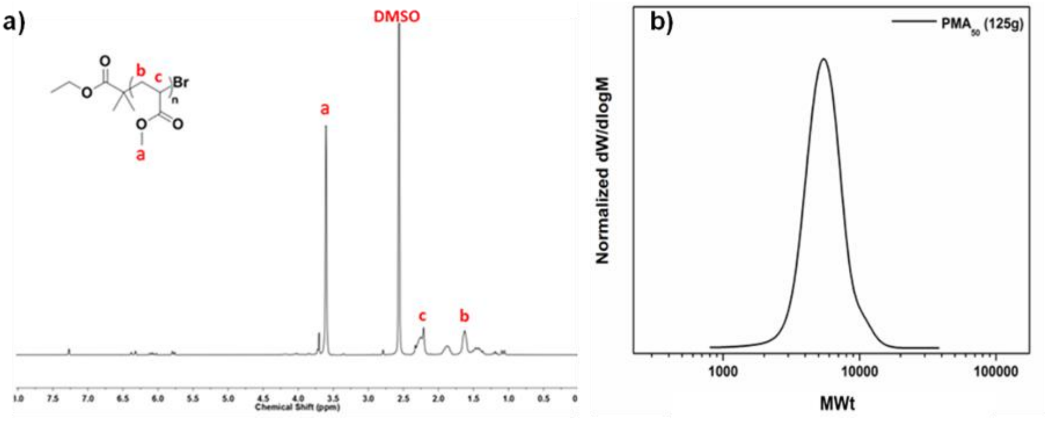

Figure S5. a) ^{1}H NMR of crude PMA_{50} in $CDCl_3$. Conversion (92%) was determined by comparing the integrals of monomeric vinyl protons (~5.7-6.5 ppm) to polymer signal, and b) THF-SEC trace of PMA_{50} (synthesis performed at RT) with M_n= 5200^a, $Đ$ =1.10, [MA]:[EBiB]:[$CuBr_2$]:[Me_6Tren] = [50]:[1]:[0.05]:[0.18]. DMSO as solvent 50% *v/v*. ([a] Molecular Weight equivalents to PMMA). The polymer was synthesised on a 250 mL scale with 125g of polymer obtained.

Table S2. Cu(0)-mediated RDRP of MA targetting a range of DP_n values (in the absence of any external deoxygenation).

DP	Conversion (%)	$M_{n,\ th.}$ (g/mol)	$M_{n,\ SEC}$ [a]	$Đ$
100	97	8500	11700	1.09
200	95	16500	20000	1.16
400	91	31500	35000	1.09
600	90	46700	48200	1.11
1000	89	79400	87000	1.13

[a] Molecular Weight equivalents to PMMA.

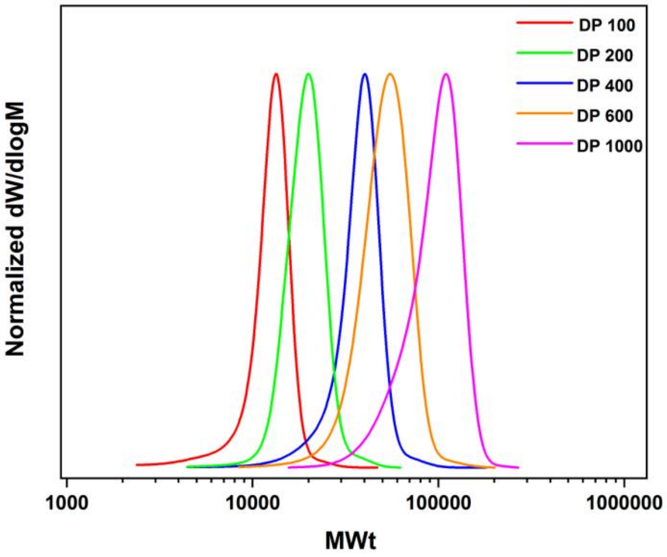

Figure S6. SEC traces for PMA with DPs 100-1000.

Table S3. ^{1}H NMR and SEC analysis for the Cu(0)-mediated RDRP of MA with DP_n=50 in different solvents (in the absence of external deoxygenation).

Solvent	Conversion (%)	$M_{n,\ th.}$ (g/mol)	$M_{n,\ SEC}$ [a]	$Đ$
ACN	>99	4500	5600	1.11
IPA	95	4300	4900	1.09
80%Tol-20%MeOH	98	4400	4900	1.10
TFE	>99	4500	5200	1.07

[a] Molecular Weight equivalents to PMMA.

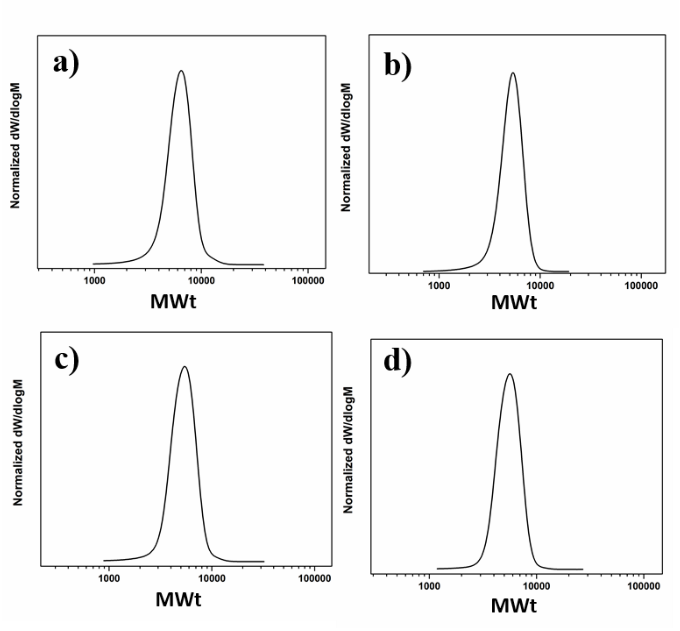

Figure S7. SEC traces of PMA_{50} in a) ACN, b) IPA, c) 80% toluene - 20% methanol and d) TFE

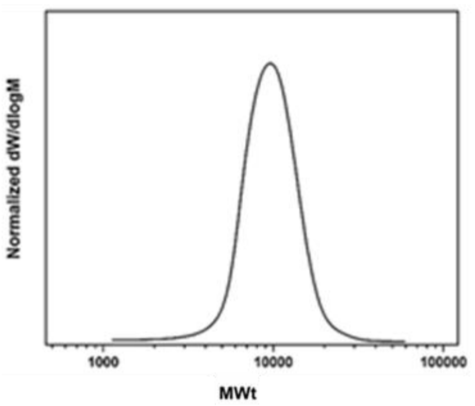

Figure S8. SEC trace of poly(*n*-BA)$_{50}$ (synthesis performed at RT) yielding M_n= 8800[a], $Đ$ =1.16, 99% conversion. [BA]:[EBiB]:[$CuBr_2$]:[Me_6Tren] = [50]:[1]:[0.05]:[0.18]. DMSO as reaction solvent 50% *v/v.* ([a] Molecular Weight equivalents to PMMA.)

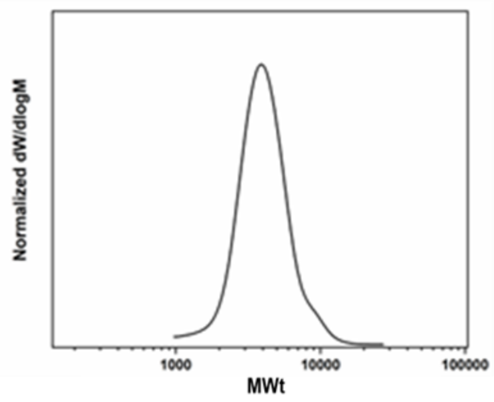

Figure S9. SEC trace of poly($PEGA_{480}$)$_{10}$ with M_n= 3700^a, $Đ$ =1.18, 82% conversion. [$PEGA_{480}$]:[EBiB]:[$CuBr_2$]:[Me_6Tren] = [50]:[1]:[0.05]:[0.18]. H_2O as reaction solvent 50% *v/v.*(^{a} Molecular Weight equivalents to PMMA).

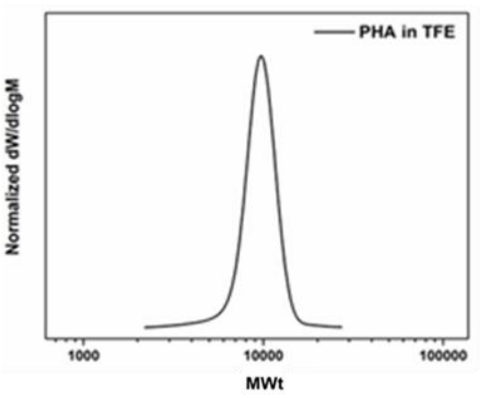

Figure S10. SEC trace of poly(HA)50 with M*n*= 9000^a, Đ =1.07, 99% conversion. [HA]:[EBiB]:[CuBr2]:[Me6Tren] = [50]:[1]:[0.05]:[0.18]. TFE reaction solvent 50% *v/v*. (^{a} Molecular Weight equivalents to PMMA).

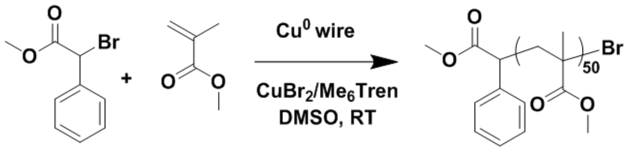

Scheme S2: Reaction scheme for the Cu(0)-mediated RDRP of PMMA$_{50}$.

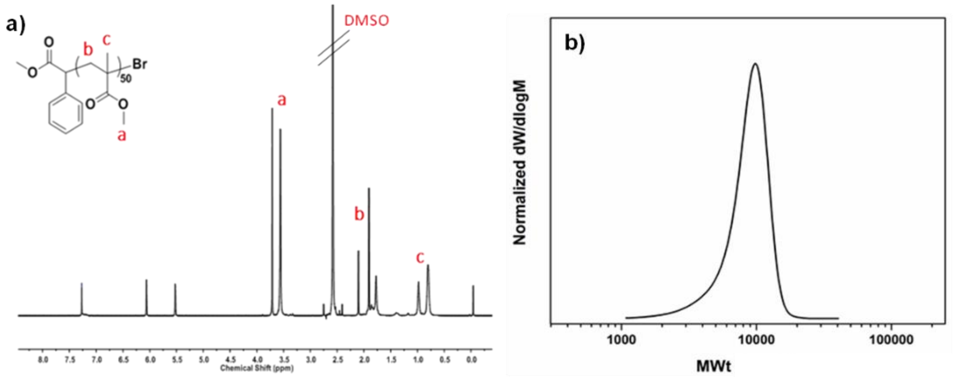

Figure S11. a) ^{1}H NMR of PMMA in $CDCl_3$. Conversion calculated by integration of methyl protons (**a**) with vinyl protons at ~6 ppm (77% conversion) and **b)** THF-SEC trace of poly(MMA)$_{50}$ with M_n= 7800^a and $Đ$ =1.15. [MMA]:[MBPA]:[$CuBr_2$]:[Me_6Tren] = [50]:[1]:[0.05]:[0.18]. DMSO solvent 50% *v/v.* (^{a} Molecular Weight equivalents to PMMA).

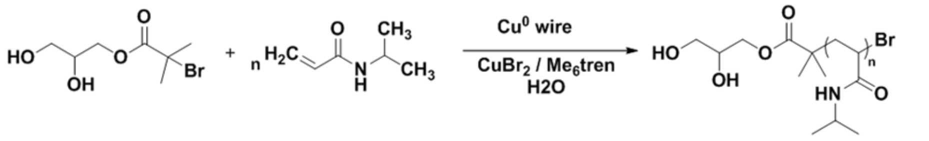

Scheme S3. Reaction scheme for the Cu(0)-mediated RDRP of NiPAm with targeted DP$_n$=50.

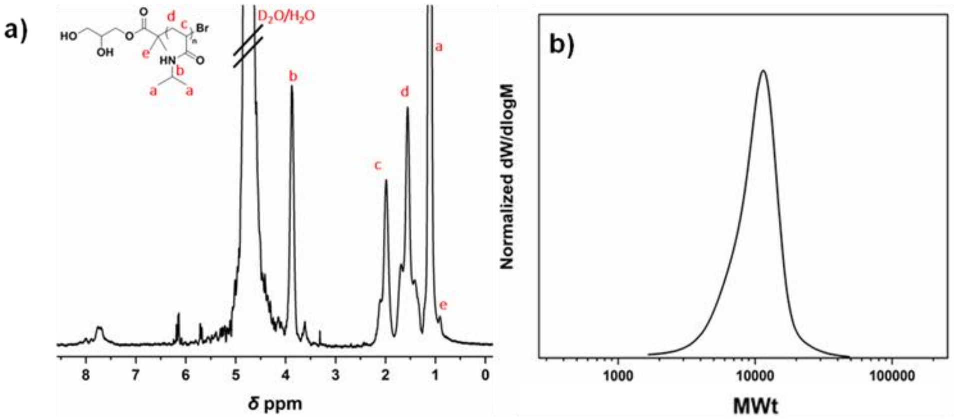

Figure S12. a) ^{1}H NMR of crude PNiPAm$_{50}$ in D_2O. Conversion calculated by integration of vinyl groups at 5.6 - 6.2 ppm with the isopropyl proton at 3.9 ppm and **b)** DMF-SEC trace of poly(NiPAm)$_{50}$ with M_n= 9000[a], $Đ$ =1.19 and conversion >99%. [NiPAm]:[I]:[$CuBr_2$]:[Me_6Tren] = [50]:[1]:[0.2]:[0.4]. ([a] Molecular Weight equivalents to PMMA).

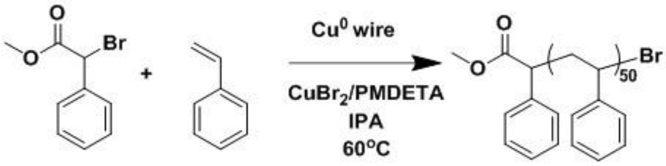

Scheme S4: Reaction scheme for the Cu(0)-mediated RDRP of PS$_{50}$.

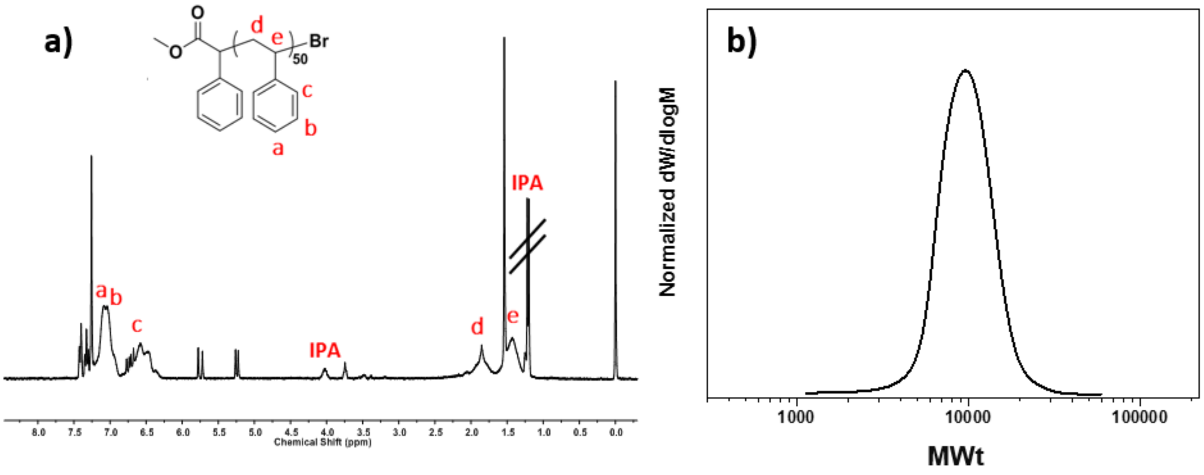

Figure S13. a) ^{1}H NMR of crude PS$_{50}$ in $CDCl_3$ yielding conversion of 91% and **b)** THF-SEC trace of PS$_{50}$ with M_n= 5400 g/mol[a], $Đ$ =1.20, with a ratio of [St]:[I]:[$CuBr_2$]:[PMDETA] = [50]:[1]:[0.05]:[0.36] performed in IPA at 60°C. ([a] Molecular Weight based on PSt standards).

Table S4. ^{1}H NMR and SEC analysis for the *in situ* chain extensions.

Block	Deoxygenation Process	Conversion (%)	$M_{n, SEC}{}^{a}$	*Đ*
PMA_{50}	None	97	6200	1.05
PMA_{50}-PMA_{50}		76	12900	1.06
PMA_{50}	2nd monomer aliquot deoxygenated	97	6300	1.06
PMA_{50}- PMA_{50}		95	12600	1.08
PMA_{50}	None	99	6500	1.08
PMA_{50}- PHA_{50}		76	14800	1.10

[a] Molecular Weight equivalents to PMMA

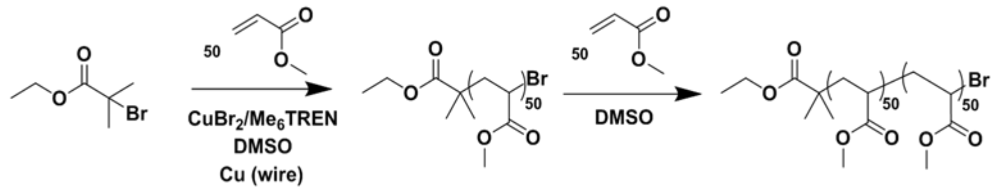

Scheme S5: Reaction scheme for the *in situ* chain extension of PMA_{50} *via* Cu(0)-mediated RDRP.

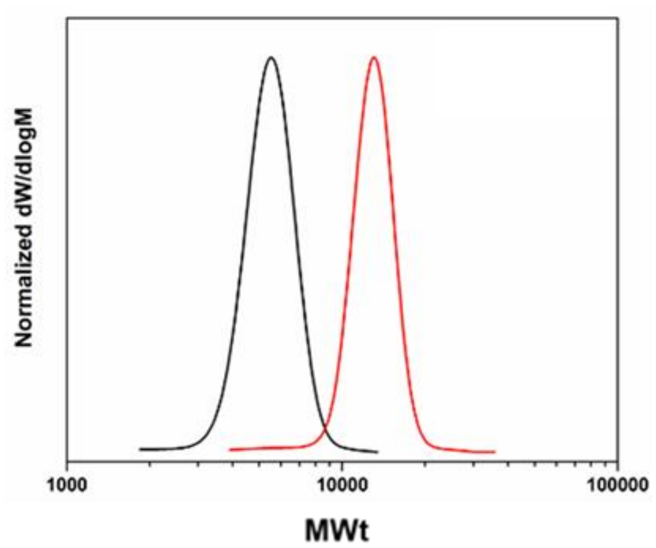

Figure S14: SEC traces of the *in situ* chain extension of PMA_{50}-PMA_{50} in DMSO with the second aliquot of MA deoxygenated *via* nitrogen sparging.

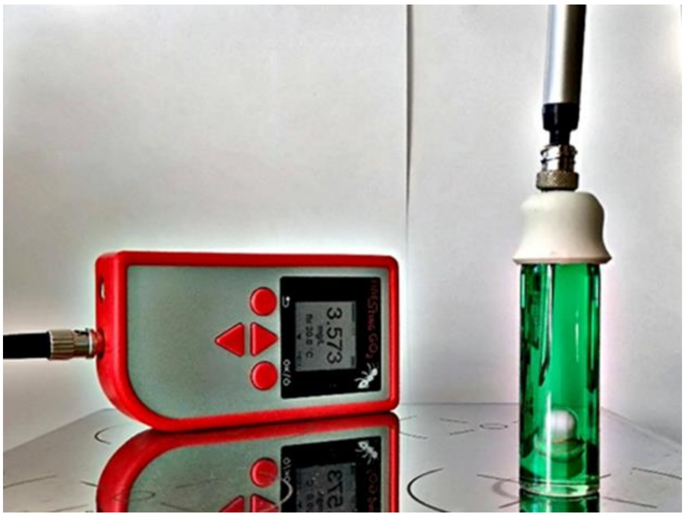

Figure S15: Setup of the *in situ* oxygen monitoring for a typical, not externally degassed Cu(0)-mediated RDRP.

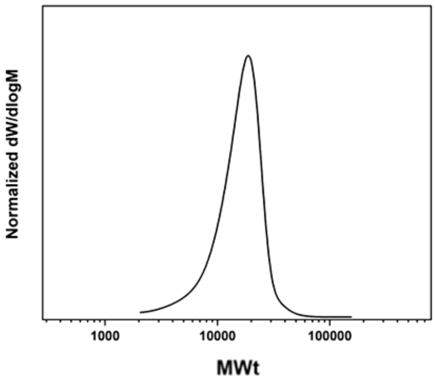

Figure S16: SEC trace of $PMMA_{100}$ *via* normal ATRP (synthesis performed at 90ºC) in toluene with M_n= 13,100[a], *Đ* ~1.2, 97% conversion. ([a] Molecular Weight equivalents to PMMA).

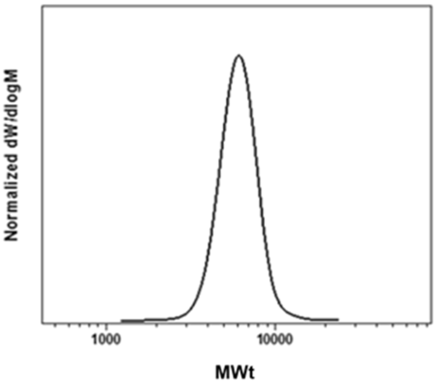

Figure S17: SEC trace for PMA_{50} obtained *via* photo-mediated RDRP in the absence of external deoxygenation with M_n ~ 5900^a, $Đ$ ~ 1.08, 98% conversion, with [EBiB]: [MA]: [$CuBr_2$]: [Me_6Tren] = 1: 50: 0.02: 0.12 and DMSO 50% *v/v* as solvent. (^{a} Molecular Weight equivalents to PMMA).

References

[1] Ciampolini, M.; Nardi, N., *Inorganic Chemistry* **1966,** *5* (1), 41-44.

[2] Haddleton, D. M.; Crossman, M. C.; Dana, B. H.; Duncalf, D. J.; Heming, A. M.; Kukulj, D.; Shooter, A. J., *Macromolecules* **1999,** *32* (7), 2110-2119.

[3] Perrier, S.; Armes, S. P.; Wang, X. S.; Malet, F.; Haddleton, D. M., *Journal of Polymer Science Part A: Polymer Chemistry* **2001,** *39* (10), 1696-1707.

Author Contributions

E.L. & R.W.: main investigation: equal
D.M.H & A.A. corresponding authors: equal
G.R.J, N.G.E, K.V.: investigation: supporting
